# Supplementary material for: Splice-Junction-Based Mapping of Alternative Isoforms in the Human Proteome
Source: Cell Rep. Author manuscript; Available in PMC 2020 Jan 15. (PMC6961840; doi:10.1016/j.celrep.2019.11.026)
Supplement: 2 [file NIHMS1546469-supplement-2.zip › DF1/PXD009737/TestisLysC/TestisLysC_5_ESPN_ASRELPPPPPPPPPPLPEAASSPPPAPPLPLESAGPGCGQRRSSSSTGK.pdf]

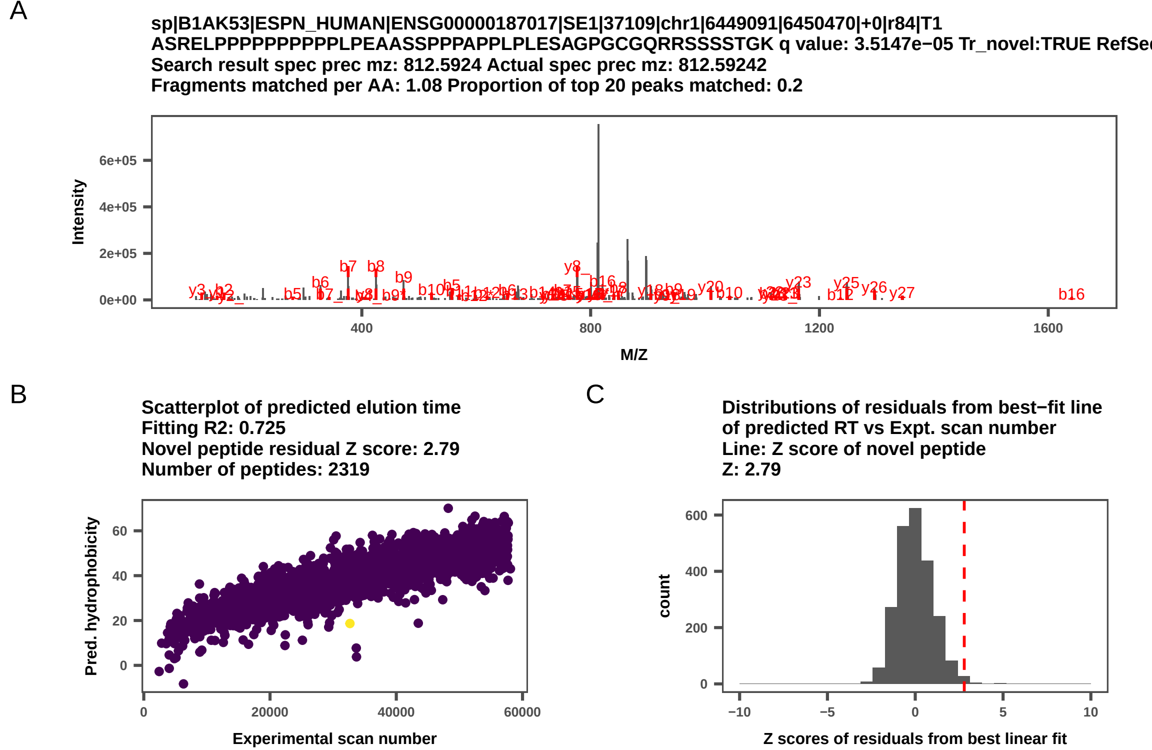
A
sp|B1AK53|ESPN_HUMAN|ENSG00000187017|SE1|37109|chr1|6449091|6450470|+0|r84|T1
ASRELPPPPPPPPPPLPEAASSPPPAPPLPLESAGPGCGQRRSSSSTGK q value: 3.5147e−05 Tr_novel:TRUE RefSe
Search result spec prec mz: 812.5924 Actual spec prec mz: 812.59242
Fragments matched per AA: 1.08 Proportion of top 20 peaks matched: 0.2
Intensity
6e+05
4e+05
2e+05
0e+00
M/Z
400
800
1200
1600
b7
b8
b9
y8
b16
y20
y23
y25
y26
y27
b16
B
Scatterplot of predicted elution time
Fitting R2: 0.725
Novel peptide residual Z score: 2.79
Number of peptides: 2319
Pred. hydrophobicity
60
40
20
0
0
20000
40000
60000
Experimental scan number
C
Distributions of residuals from best−fit line
of predicted RT vs Expt. scan number
Line: Z score of novel peptide
Z: 2.79
count
600
400
200
0
−10
−5
0
5
10
Z scores of residuals from best linear fit
